# Effect of increasing levels of phytase on performance, prececal nutrient digestibility, intestinal mucosa physiology and immune response in broiler chickens from 1 to 21 days of age^☆^

**DOI:** 10.1016/j.psj.2025.105473

**Published:** 2025-06-24

**Authors:** B. Martínez-Vallespín, P. Ader, J. Zentek

**Affiliations:** a Institute of Animal Nutrition, Department of Veterinary Medicine, Freie Universität Berlin, Königin-Luise-Str. 49, 14195 Berlin, Germany; b BASF SE, 68623 Lampertheim, Germany

**Keywords:** Phytase, Broiler, Performance, Apparent ileal digestibility, Intestinal physiology

## Abstract

Phytic acid is the primary storage form of phosphorus (P) in plants and broiler chickens have a limited capability for its utilization. The supplementation of exogenous phytase in feed enables broilers to use the phytate-bound P. The current study evaluated the supplementation of increasing levels of a bacterial hybrid 6-phytase (500, 1,500 and 3,000 FTU/kg feed; groups LP2, LP3 and LP4, respectively) to a low-P, phytate-containing broiler chicken diet (LP1), fed from day 1 to 21 of age. Statistical analysis used a general linear model with linear and quadratic contrasts to assess phytase-level trends. The efficacy of the phytase on performance and ileal digestibility was demonstrated with linear increases in body weight (BW), BW gain, feed intake and apparent ileal digestibility of most of the amino acids, as well as of P (*P* < 0.05). The increasing levels of phytase led to a linear decrease of jejunal and caecal crypt depth when corrected for BW (*P* < 0.05), and to a linear decrease of the villus height:crypt depth ratio (*P* = 0.028). Secretory IgA measured in jejunum digesta showed a trend to a linear increase with the increasing levels of phytase (*P* = 0.092). The measurement of d-glucosamine and d-galactosamine as markers of mucus secretion in ileum digesta showed differences for d-glucosamine only, with a linear increase of this marker due to the phytase supplementation (*P* = 0.017). Finally, a trend for increased *ex situ* glucose transport was observed in Ussing chambers when tissue of the jejunal mucosa of LP1 group was compared with that of LP4 group (*P* = 0.079). Quadratic effects suggest a stabilizing response in most traits beyond a certain phytase level, indicating a biological plateau. In conclusion, the use of increasing doses of exogenous bacterial hybrid 6-phytase up to 3,000 FTU/kg in broilers from day 1 to 21 of age improved performance and nutrient digestibility as well as some traits related to intestinal physiology and immune response.

## Introduction

Phytic acid (myo-inositol 1,2,3,4,5,6-hexakis dihydrogen phosphate; **IP_6_**) is a naturally occurring compound in plants and the main molecule of phosphorus (**P**) storage in plant-based ingredients used in broiler diets (Bedford, 2000). However, broiler chickens have a limited ability to utilize this P. While some studies suggest that broilers may have relatively efficient endogenous phytases in the prececal region, these enzymes have not yet been thoroughly characterized (Rodehutscord et al., 2022). Moreover, different factors such as the level of inorganic P and calcium (**Ca**) in the diet or the commercial strains seem to influence the efficiency of these endogenous enzymes.

The supplementation of microbial-derived exogenous phytases became an established strategy to release the phosphate groups from the inositol ring by hydrolysis that leads to metabolites with 5, 4, 3, 2, 1 or even 0 remaining P called IP5, IP4, IP3, IP2, IP1 and IP0, respectively (Krieg et al., 2020). For more than three decades, phytases have regularly been used as feed additives in broiler feeds as a strategy for improving plant-derived P digestibility. At the same time, the amount of inorganic P in poultry diets could be reduced and resulted in a markable decrease of P excretion (Józefiak et al., 2016). Furthermore, IP_6_ is known to bind to other feed compounds like minerals or proteins, forming insoluble complexes and hereby reducing the utilization of these nutrients by the animal (Singh, 2008; Bedford and Walk, 2016).

In previous studies it has been indicated that the beneficial effects of phytase supplementation are potentially not limited to P-release only (Cowieson et al., 2011). The so-called “superdosing”, meaning the addition of high phytase doses beyond the purpose of replacing dietary inorganic P only, resulted in positive effects on performance indicators and effects have been found for being dose-dependent to some extent (Kies et al., 2006; Walters et al., 2019; Martínez-Vallespín et al., 2022).

It is suggested that IP_6_ may directly or indirectly affect the physiology of the intestinal mucosa by causing mucus secretion (Onyango et al., 2009). Some studies have measured the effect of the addition of phytase to phytate-containing diets on intestinal morphology but the reported results are not consistent (Wu et al., 2004; Emami et al., 2013; Nari et al., 2020; Moita et al., 2021). A recent study has shown that the use of a bacterial phytase in young broilers led to a decrease in crypt depth in both jejunum and cecum, and was interpretated as a possible sign of lower cell renewal due to lower epithelial challenge by the IP_6_ (Martínez-Vallespín et al., 2022). Moreover, the birds fed with phytase supplemented diets have shown lower counts of CD3-positive intraepithelial lymphocytes in cecal tissue compared to the non-supplemented control, while counts decreased with increasing levels of dietary phytase (Martínez-Vallespín et al., 2022).

There is still limited information on the effects of phytase-supplemented feed on mechanisms related to intestinal epithelial physiology, as well as on the question of effects independent of phosphorus (P), the so-called 'extra-phosphoric effects', such as mucosal absorption and secretion mechanisms, and impacts on gut-related immune responses. Thus, the purpose of the study was to evaluate in more detail the effects of increasing levels of dietary phytase on mucosal morphology and related physiological and immunological responses in broiler chickens at 21 days of age.

## Materials and methods

This study was performed in accordance with the Animal Welfare Act of Germany once approved by the State Office of Health and Social Affairs Berlin (Landesamt für Gesundheit und Soziales, LaGeSo; No. 0439/17).

### Animals and experimental design

A total of 320 one-day-old healthy male broiler chickens (Cobb 500) were obtained from a local hatchery (Cobb Germany Avimex GmbH, Wiedemar, Germany). The birds were vaccinated against Infectious Bronchitis (IB Primer: Zoetis) and Newcastle Disease (ND: Merial AviNew). After arrival, chickens were randomly allocated to 40 floor pens within a climate-controlled poultry house using eight birds per pen (eight repetitions per treatment group). The poultry house was provided with artificial light (30 lux), a controlled climate, and forced ventilation (0.4–0.5 m^3^/h/kg body weight). The barn was pre-warmed for 48 h prior to poultry placement and the temperature was kept at 32°C during the first week of the trial being gradually diminished to 29°C and 26°C during the second and third weeks, respectively. The artificial light was kept on for 24 h during the four first days and from day 5 the lighting regime consisted of an 18 h light and 6 h dark cycle. Birds had ad libitum access to feed and water throughout the whole experiment.

The trial lasted 21 days and during the whole experimental period, one basal diet in pelleted form were used. A basal phytate-containing diet with low P level (LP1) based on corn, soybean meal and sunflower meal was formulated. The LP1 diet was supplemented with increasing dose rates (500, 1500 and 3000 FTU/kg) of a bacterial hybrid 6-phytase (NATUPHOS E, BASF SE, Ludwigshafen, Germany) for obtaining the diet LP2, LP3 and LP4 (Table 1). The diets were formulated to meet or slightly exceed the nutrient requirements for broiler chicken recommended by the Society of Nutritional Physiology (GfE, 1999) with the exception of metabolizable energy (ME), crude protein (CP), amino acids (AA) and P, which were slightly reduced. The Ca content was in accordance with the recommended minimum specifications given by the breeder (9 g/kg). NATUPHOS E supplementation was done at the expense of Tixosil (silicon dioxide >97 %; Solvay GmbH, Hannover, Germany). In addition, Titanium (IV)-dioxide (TiO_2_) was added as a marker for apparent ileal digestibility (AID) measurements at a dose level of 3 g/kg.

**Table 1:** Ingredients and nutritional characteristics of the experimental diets (as is).

		LP1^1^	LP2	LP3	LP4
Phytase level	(FTU/kg feed)		500	1,500	3,000
		Ingredients			
Corn	%	54.1	54.1	54.1	54.1
Soybean meal (49 %)	%	26.1	26.1	26.1	26.1
Sunflower meal	%	12.0	12.0	12.0	12.0
Soybean-oil	%	3.87	3.87	3.87	3.87
Limestone	%	1.93	1.93	1.93	1.93
Monocalcium-phosphate	%	0.21	0.21	0.21	0.21
Premix 2	%	1.20	1.20	1.20	1.20
L-Lysine – HCL	%	0.13	0.13	0.13	0.13
DL-Methionine	%	0.09	0.09	0.09	0.09
L-Tryptophan	%	0.04	0.04	0.04	0.04
Titanium dioxide	%	0.30	0.30	0.30	0.30
Tixosil 3	%	0.05	0.04	0.02	-
Natuphos® E 5000 G	%	-	0.01	0.02	0.05
		Calculated analysis			
ME 4	kcal/kg	2,964	2,964	2,964	2,964
Crude protein	%	22.3	22.3	22.3	22.3
Lysine	%	1.20	1.20	1.20	1.20
Methionine	%	0.46	0.46	0.46	0.46
Methionine & Cystine	%	0.84	0.84	0.84	0.84
Threonine	%	0.84	0.84	0.84	0.84
Tryptophan	%	0.29	0.29	0.29	0.29
Crude fat	%	6.74	6.74	6.74	6.74
Crude fiber	%	4.48	4.48	4.48	4.48
Crude ash	%	5.21	5.21	5.21	5.21
Calcium	%	0.90	0.90	0.90	0.90
Phosphorus	%	0.50	0.50	0.50	0.50
Sodium	%	1.80	1.80	1.80	1.80
		Analyzed composition			
Dry matter	g/kg	904	902	903	900
Crude protein	g/kg	211	217	213	217
Crude fiber	g/kg	53.7	52.8	55.9	50.1
Crude ash	g/kg	54.0	52.5	52.7	53.9
Crude fat	g/kg	61.3	64.7	63.0	63.1
Starch	g/kg	380	370	374	372
Total sugars	g/kg	39	40	42	44
Calcium	g/kg	8.70	8.02	7.88	8.08
Phosphorus	g/kg	4.9	4.8	4.8	4.9
Sodium	g/kg	1.72	1.53	1.50	1.61
Phytase	FTU/kg	180	618	1,579	2,644
Phytate phosphorus	g/kg	2.54	2.54	2.54	2.54

1 LP1: low-phosphorus diet; LP2: low-phosphorus diet + 500 FTU/kg feed; LP3: low-phosphorus diet + 1,500 FTU/kg feed; LP4: low-phosphorus diet + 3,000 FTU/kg feed.

2 Contents per kg: 600,000 I.U. Vit. A (retinyl-acetate); 120,000 I.U. Vit. D_3_; 6,000 I.U. Vit. E (α-tocopherol acetate); 200 mg Vit. K_3_ (MSB); 250 mg Vit. B_1_ (mononitrate); 420 mg Vit. B_2_ (cryst. riboflavin); 300 mg Vit. B_6_ (pyridoxin-HCl); 1500 μg Vit. B_12_; 3,000 mg niacin (niacinamide); 12,500 μg biotin (commercial, feed grade); 100 mg folic acid (cryst., commercial, feed grade); 1,000 mg pantothenic acid (Ca d-pantothenate); 60,000 mg choline (chloride); 5,000 mg iron (iron carbonate); 5,000 mg zinc (zinc sulfate); 6,000 mg manganese (manganous oxide); 1,000 mg copper (copper oxide); 45 mg iodine (calcium-iodate); 20 mg selenium (sodium-selenite); 140 g sodium (NaCl); 55 g magnesium (magnesium sulfate); carrier: calcium carbonate (calcium min 38 %).

3 Silicon dioxide > 97 %.

4 Estimated according to equation of WPSA 1984 (by using crude nutrients).

All broiler chickens were observed twice daily for any abnormalities, abnormal behavior, and clinical signs of sickness throughout the trial. Morbidity and mortality were also recorded.

### Growth performance

Body weight (BW) and feed consumed were recorded weekly and body weight gain (BWG), feed intake (FI) and feed conversion ratio (FCR) were calculated. The European Poultry Efficiency Factor (EPEF), which is a value that standardizes technical results, considering FCR, mortality/culling, and BW was also calculated.

### Sample collection

On day 21, two birds per box were euthanized for sample collection. In addition, 8 animals were euthanized on days 21, 22, 23 and 26 for jejunal tissue collection for Ussing chamber studies. The birds were stunned and killed by exsanguination.

Ileal digesta from the posterior half between Meckel’s diverticulum and 2 cm before the ileo-ceco-colonic junction was collected for digestibility and mucus production measurements. Due to the low amount, the ileal digesta of the two slaughtered animals per box was pooled. The AID of nutrients was calculated using the following formula:

$$AID\;\left(\%\right)=100-\left(\frac{\%\;marker\;in\;feed}{\%\;marker\;in\;ileum}\times \frac{\%\;nutrient\;in\;ileum}{\%\;nutrient\;in\;feed}\right)\times 100$$

For all other samples, only one animal was used. Jejunal and cecal tissue samples were collected and preserved in formalin for morphological measurements as well as for goblet cells. Jejunal digesta was collected for IgA measurements.

### Histological procedures

The jejunal and cecal sections were initially fixed in 4 % phosphate-buffered formaldehyde (Carl Roth GmbH, Karlsruhe, Germany). Later, they were dehydrated in increasing concentrations of ethanol (from 70 % to 96 %) and isopropanol, and cleaned with xylol and immediately after, they were embedded in paraffin wax. Five µm sections were cut with a sledge microtome (Type 1400, Leitz, Wetzlar, Germany), mounted on glass slides, and dried in an incubator at 37°C. Before staining, the sections were deparaffinized with xylol and rehydrated with decreasing concentrations of ethanol. Hematoxylin/eosin staining was performed allowing the measurement of the villus height (jejunum) and crypt depth (jejunum and cecum) and Alcian blue-periodic acid Schiff staining was used for goblet cell count. A light microscope (Olympus BX43, Olympus Co., Tokyo, Japan) equipped with a digital camera (DP72, Olympus, Hamburg, Germany) was used. The measurements were taken using cellSens imaging software (v. 1.4, Olympus, Hamburg, Germany).

### Immunoglobulin A

Secretory IgA was measured in jejunal digesta by enzyme-linked immunosorbent assay (chicken IgA ELISA kit ab157691, Abcam, Cambridge, UK) according to the manufacturer’s instructions.

### Ussing chamber studies

For this method, only groups LP1 and LP4 were compared. The entire jejunum was resected immediately after slaughter, transferred into an ice-cold carbogen-gassed experimental transport buffer modified Krebs-Henseleit buffer (115 mM NaCl 25 mM NaHCO_3_, 5 mM KCl, 2.4 mM Na_2_HPO_4_, 1.5 mM CaCl_2_, 1.2 mM MgCl_2_, 0.6 mM NaH_2_PO_4_, 10 mM glucose, 2 mM mannitol, pH 7.4, ∼320 mOsmol/l) and brought immediately to the laboratory. The intestinal tube was cut open longitudinally on its mesenteric side and rinsed. The epithelium was stripped of the serosa and muscle layers and immediately mounted in the Ussing chambers provided with a supporting net and with an exposed area of 0.79 cm^2^ (Ruhnke et al., 2013). The apical and basolateral side of the tissue were bathed in 15 ml of a buffer solution at 38°C through surrounding water-jacketed reservoirs. The buffer in the chambers was similar to the transport buffer without glucose and with 2 mM of Mannitol. Continuous gassing with carbogen was provided. Electrical measurements were obtained by a microcomputer-controlled voltage/current clamp (K. Mussler Scientific Instruments, Aachen, Germany). The transepithelial potential difference in response to bipolar 50 μA current pulses generated for 200 ms and the tissue conductance (Gt) was calculated every 6 sec by Ohm’s law. After equilibration for approximately 15-30 min, tissues were short-circuited by clamping the voltage at 0 mV. After reaching the baseline with constant values of the short-circuit current (**Isc**), active transport in the tissue samples was studied by adding in the first place a 10 mM glucose solution to the mucosal side. To keep the same osmolarity in the buffer in both sides of the tissue, a 10 mM solution of mannitol was added to the serosal side. Finally, and after again reaching constant values of the Isc, the tissue sample was exposed to 100 μM carbachol in order to evaluate the secretory capacity of the jejunum. Carbachol was added to the serosal buffer solution. All chemicals were added without removing the previous substances. All chemicals were purchased from Sigma-Aldrich (Schnelldorf, Germany).

### Chemical analyses

All experimental diets were ground to pass through a 0.25 mm screen before analysis. Laboratory measurements included Weende constituents and, additionally, AA, starch, total sugars, Ca and P. Analyses were in accordance with the methods issued by VDLUFA (dry matter: VDLUFA III 3.1; CP: VDLUFA III 4.1.2 modified according to macro-N determination (vario MAX CN); AA: VDLUFA III 4.11.1; crude fiber: VDLUFA III 6.1.4; crude ash: VDLUFA III 8.1; crude fat: VDLUFA III 5.1.1; starch: VDLUFA III 7.2.1; total sugars: VDLUFA III 7.1.1; Ca and P: VDLUFA VII 2.2.2.6). Samples were also analyzed to confirm the concentrations of phytase activity (ISO 30024:2009) and phytate P content (Haug and Lantzsch, 1983). Pooled ileal samples were ground to pass through a 0.25 mm screen and analyzed for crude ash, Ca, P, and AA.

For mucus production, d-galactosamine and d-glucosamine were analyzed, as they are major components of mucus and can be used as markers of the mucus production in the gut. The analytical technique begins with a preliminary deacetylation stage, followed by hydrolysis of the sample to yield d-galactosamine and d-glucosamine. Both of them were measured using the same method as for AA.

### Statistical analysis

Statistical analyses were performed using the SPSS software package (Version 25; IBM, Chicago, IL, USA). A general linear model was applied, and additional linear and quadratic contrasts were conducted to evaluate potential trends across increasing levels of phytase. The cage was considered the experimental unit for performance traits; for all other measured variables, the individual animal served as the experimental unit. Differences were considered statistically significant at *P* < 0.05, while mean differences with *P*-values between 0.05 and 0.10 were interpreted as trends.

## Results

### Animal health and performance

Broiler chickens were healthy during the study. The overall mortality rate, with the inclusion of culling, amounted to 2.5 %.

Animals in all groups had a similar BW at day 1, averaging 43.6 ± 1.23 g (Table 2). Supplementation of LP1 with increasing levels of phytase led to improvement in BWG and, consequently, to higher BW at the end of the trial (*P* < 0.001). BW at the end of the trial, BWG, FI and EPEF increased linearly with increasing levels of phytase supplementation (*P* < 0.001). FCR decreased linearly (*P* = 0.028). All traits showed a quadratic effect, except for FCR, which exhibited only a trend (*P* < 0.05 and *P* = 0.069, respectively).

**Table 2:** Effect of different inclusion levels of phytase on growth performance of broiler chickens throughout the experimental period of 21 days.

								Contrasts	
		LP1^1^	LP2	LP3	LP4	SEM^2^	*P*-value	Linear	Quadratic
Body weight									
- start	g	44.0	43.6	42.9	43.8	0.21	0.305		
- end	g	595	655	794	885	22.7	< 0.001	< 0.001	0.001
Body weight gain	g	551	611	751	841	22.7	< 0.001	< 0.001	0.001
Body weight gain	g/day	27.5	30.6	37.6	42.1	1.13	< 0.001	< 0.001	0.001
Feed intake	g	750	871	1039	1115	28.6	< 0.001	< 0.001	< 0.001
Feed intake	g/day	37.5	43.6	52.0	55.8	1.43	< 0.001	< 0.001	< 0.001
Feed conversion ratio	g/g	1.36	1.43	1.38	1.33	0.01	0.011	0.016	0.069
EPEF^3^		191	215	268	312	9.75	< 0.001	< 0.001	0.018

1 LP1: low-phosphorus diet; LP2: low-phosphorus diet + 500 FTU/kg feed; LP3: low-phosphorus diet + 1,500 FTU/kg feed; LP4: low-phosphorus diet + 3,000 FTU/kg feed.

2 Standard error of the mean.

3 European Poultry Efficacy Factor.

### Apparent ileal nutrient digestibility

Increasing dose rates of phytase supplemented to LP1 led to a linear increase of AID for crude ash and P (*P* < 0.001; Table 3), whereas AID of Ca linearly decreased (*P* < 0.001). The AID of all AA linearly increased by supplementing LP1 with phytase (*P* ≤ 0.05 and *P* = 0.079 for Cys). Results also revealed a quadratic effect in all traits (*P* < 0.001).

**Table 3:** Effect of different inclusion levels of phytase on apparent ileal digestibility (%) of main nutrients and amino acids in broiler chickens at 21 days of age.

							Contrasts	
	LP1^1^	LP2	LP3	LP4	SEM^2^	*P*-value	Linear	Quadratic
Crude ash	39.5	43.0	45.7	48.5	0.99	0.004	< 0.001	< 0.001
Phosphorus	41.1	49.2	51.3	60.7	1.78	< 0.001	< 0.001	< 0.001
Calcium	55.1	55.5	47.3	43.9	1.28	< 0.001	< 0.001	< 0.001
Alanine	78.4	81.4	84.7	84.8	0.84	0.009	0.002	< 0.001
Arginine	87.2	88.8	90.2	91.7	0.50	0.003	<0.001	< 0.001
Aspartic acid	77.3	78.1	82.2	82.0	0.75	0.017	0.004	< 0.001
Cysteine	59.4	73.9	70.5	71.7	1.97	0.031	0.079	< 0.001
Glutamic acid	85.4	86.5	88.4	89.3	0.50	0.014	0.002	< 0.001
Glycine	70.8	73.4	76.9	78.0	1.04	0.023	0.004	< 0.001
Histidine	79.5	81.5	85.2	85.2	0.79	0.010	0.002	< 0.001
Isoleucine	77.9	81.2	84.2	84.4	0.94	0.022	0.006	< 0.001
Leucine	79.5	79.7	85.0	85.3	0.82	0.004	<0.001	< 0.001
Lysine	81.3	84.1	86.7	86.5	0.80	0.031	0.009	< 0.001
Methionine	83.2	90.7	90.8	91.3	0.91	<0.001	0.002	< 0.001
Phenylalanine	81.3	83.3	86.1	86.5	0.72	0.018	0.003	< 0.001
Proline	77.6	79.3	81.9	83.5	0.75	0.010	0.001	< 0.001
Serine	78.6	78.3	81.5	83.1	0.69	0.028	0.004	< 0.001
Threonine	73.4	75.2	78.6	79.3	0.88	0.033	0.006	< 0.001
Tyrosine	72.8	79.7	82.8	81.9	1.46	0.034	0.020	< 0.001
Valine	75.3	78.9	82.0	82.1	0.99	0.022	0.006	< 0.001

1 LP1: low-phosphorus diet; LP2: low-phosphorus diet + 500 FTU/kg feed; LP3: low-phosphorus diet + 1,500 FTU/kg feed; LP4: low-phosphorus diet + 3,000 FTU/kg feed.

2 Standard error of the mean.

### Histological studies

Results regarding histomorphology of jejunum and caecum are shown in Table 4. There was a clear effect of supplementing LP1 with increasing phytase levels on jejunal villus height (*P* = 0.036), but the differences disappeared when the data were expressed related to BW. On the contrary, no effect was observed on jejunal crypt depth, but when this trait was expressed related to BW, a linear decrease was observed (*P* = 0.002). The villus height to crypt depth ratio (V:C) showed a linear increase with increasing levels of phytase (*P* = 0.028). No effect on goblet cells count was observed in either the villi or the crypts.

**Table 4:** Effect of different inclusion levels of phytase on morphometry and goblet cell count in jejunum and caecum of broiler chickens at 21 days of age.

								Contrasts	
		LP1^1^	LP2	LP3	LP4	SEM^2^	*P*-value	Linear	Quadratic
**Jejunum**									
VH^3^	µm	915	983	1,175	1,279	51.7	0.036	0.006	< 0.001
VH/kg BW	µm/kg	1,434	1,392	1,365	1,406	61.9	0.988	0.616	< 0.001
CD	µm	242	247	207	212	13.1	0.620	0.380	< 0.001
CD/kg BW	µm/kg	367	353	241	233	19.3	0.002	0.002	< 0.001
V:C ratio	–	4.58	4.29	5.76	6.15	0.31	0.097	0.028	< 0.001
GC (villi)	n	51.9	49.5	50.0	52.7	1.84	0.934	0.792	< 0.001
GC (crypts)	n	72.0	82.2	71.0	83.8	2.65	0.195	0.336	< 0.001
**Caecum**									
CD	µm	250	218	211	206	6.31	0.046	0.022	< 0.001
CD/kg BW	µm/kg	388	317	249	230	14.8	< 0.001	< 0.001	< 0.001
GC	n/mm	22.7	31.1	27.0	27.2	1.10	0.053	0.558	< 0.001

1 LP1: low-phosphorus diet; LP2: low-phosphorus diet + 500 FTU/kg feed; LP3: low-phosphorus diet + 1,500 FTU/kg feed; LP4: low-phosphorus diet + 3,000 FTU/kg feed.

2 Standard error of the mean.

3 VH: villus height; CD: crypt depth; V:C: villus height to crypt depth ratio; GC: goblet cells (expressed as mm of basement membrane).

In the cecum, crypt depth decreased linearly with increasing levels of phytase supplementation, and this effect was more pronounced when adjusted for BW (*P* = 0.022 and *P* < 0.001, respectively). An increase in goblet cell number was also observed with phytase supplementation at all levels (*P* = 0.053). Again, evidence of a quadratic effect was observed for all measured traits (*P* < 0.001).

### Immunoglobulin A in jejunum digesta

**Fig. 1:**
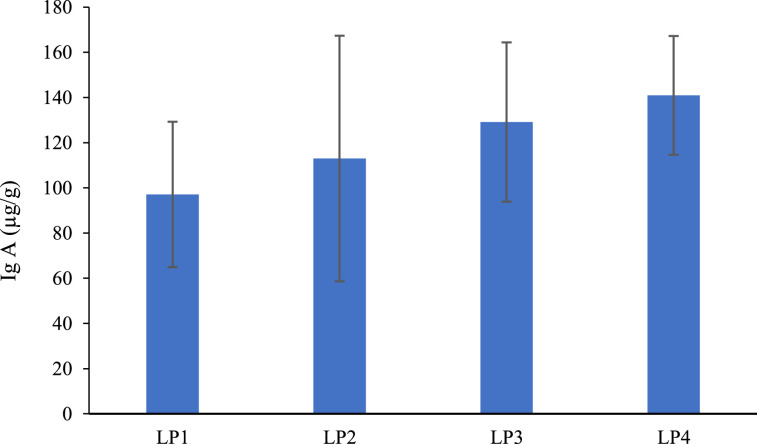
Effect of different inclusion levels of phytase on immunoglobulin A in jejunum digesta of broiler chickens at 21 days of age. LP1: low-phosphorus diet; LP2: low-phosphorus diet + 500 FTU/kg feed; LP3: low-phosphorus diet + 1,500 FTU/kg feed; LP4: low-phosphorus diet + 3,000 FTU/kg feed. *P* = 0.295. Linear contrast: *P* = 0.092. Quadratic contrast: *P* < 0.001.

IgA concentration measured in jejunal digesta is shown in Fig. 1. Increasing phytase supplementation in the LP1 diet led to a trend toward a linear increase in IgA concentration (*P* = 0.092). A significant quadratic effect was also detected (*P* < 0.001).

### Mucus markers in ileum digesta

The addition of phytase resulted in a linear increase of d-glucosamine concentration in ileal digesta (*P* = 0.017; Table 5). No differences were found for d-galactosamine concentration. Quadratic effects were observed for both traits (*P* < 0.001).

**Table 5:** Effect of different inclusion levels of phytase on mucus markers in ileum digesta of broiler chickens at 21 days of age (expressed in g/100 g digesta).

							Contrasts	
	LP1^1^	LP2	LP3	LP4	SEM^2^	*P*-value	Linear	Quadratic
D-glucosamine	0.06	0.34	0.37	0.38	0.04	0.022	0.017	< 0.001
D-galactosamine	0.04	0.05	0.06	0.06	0.01	0.313	0.124	< 0.001

1 LP1: low-phosphorus diet; LP2: low-phosphorus diet + 500 FTU/kg feed; LP3: low-phosphorus diet + 1,500 FTU/kg feed; LP4: low-phosphorus diet + 3,000 FTU/kg feed.

2 Standard error of the mean.

### Nutrient transport in jejunum tissue

The results of the Ussing chamber experiments are shown in Table 6. Following the addition of glucose, a numerical increase in Isc was recorded (*P* = 0.079). Addition of the control substance carbachol had no impact on Isc. No effects in Gt after glucose or carbachol addition were observed.

**Table 6:** Effect of d-Glucose and carbachol on short-circuit current (Isc) and transepithelial conductance (Gt) in isolated jejunal mucosa of broilers fed with selected diets (21-26 days of age).

		LP1^1^	LP4	SEM^2^	*P*-value
ΔIsc^3^ Glucose	μA/cm^2^	8.31	14.1	1.64	0.079
ΔIsc Carbachol	μA/cm^2^	8.44	8.71	1.73	0.940
ΔGt Glucose	mS/cm^2^	−0.40	−0.72	0.11	0.168
ΔGt Carbachol	mS/cm^2^	−0.06	−0.20	0.07	0.362

1 LP1: low-phosphorus diet; LP4: low-phosphorus diet + 3,000 FTU/kg feed.

2 Standard error of the mean.

3 Δ: change.

## Discussion

The current study focused on the effect of increasing levels of phytase on mucosal morphology and related physiological and immunological responses in broiler chickens at 21 days of age. The experiment was planned as a continuation of a previous study to try to confirm the findings as well as to explore further effects of the phytase (Martínez-Vallespín et al., 2022). The effects on performance of the increasing phytase concentrations were comparable in both trials. Thus, the addition of increasing doses of phytase improved performance indicators in a linear way. As discussed in our previous publication, this observation is in line with previous studies using similar phytase levels (Adejumo et al., 2021; Kriseldi et al., 2021).

The addition of increasing levels of phytase linearly increased AID of P and crude ash. However, AID of Ca decreased linearly. According to Li et al. (2017), the undigested IP esters can bind with divalent cations like Ca^+^ in the small intestine, where pH is 6 and above. Thus, the higher phytase levels potentially release more P and thereby reduce the potential for this complexation. It can be speculated that the concentrations of Ca and released P exceeded the solubility product in the small intestinal digesta, leading to the formation of insoluble complexes and thereby negatively affecting Ca utilization.

The increase in AID of AA is a common observation in studies using exogenous phytase in phytate-containing diets, although this effect is not always observed for the same AA (Ravindran et al., 2000; Rutherfurd et al., 2004; Cowieson et al., 2017; Krieg et al., 2020; Martínez-Vallespín et al., 2022). This could be attributed to the different feed compositions, phytase origins and levels. However, we have no explanation for the rather low impact on cysteine´s AID in the present study since, in general, and as reported in a previous study (Martínez-Vallespín et al., 2022) the AID of this AA was strongly improved.

The effect of supplemented exogenous phytase on intestinal morphology is not well studied, and no clear conclusions can be drawn from the literature. Moreover, a well-established association between histological measurements and performance in broilers has yet to be demonstrated. However, based on the authors´ previous observations and findings reported in the literature suggesting potential correlations (Rysman et al., 2023), it seems appropriate to correct for BW when the different treatments lead to noteworthy differences in growth performance. For example, in absolute terms, there seemed to be a clear effect of the phytase addition on jejunal villus height that disappeared after correcting for BW. On the opposite, a linear effect of the increasing levels of phytase appeared in jejunal crypt depth when data were corrected by BW.

A higher crypt depth is an indicator of an increased cell renewal due to epithelial damage, which is also reflected in a low V:C ratio (Ducatelle et al., 2018). There seems to be a disruption on the jejunal mucosa in LP1 and LP2 in comparison with LP3 and LP4 that might be explained by the presence of potentially highest levels of IP esters with high P content like IP6, IP5, IP4 in the unsupplemented and low phytase diets compared to higher phytase dose rates (Krieg et al., 2020). The V:C ratio can also indicate a more efficient absorptive function (Paiva et al., 2014) that would potentially be improved with the increasing levels of phytase according to the present data. This observation seems to be reinforced by the results observed in the cecum.

Findings are in line with previous studies where phytase dose rates between 500 and 4,000 FTU/kg feed in low P diets are reported to improve epithelial structure in small intestine, mainly by increasing villus height (Emami et al., 2013; Moita et al., 2021; Selim et al., 2022). On the contrary, few studies have not found an effect on intestinal morphology (Van Emmenes et al., 2018; Mohammadi Ziarat et al., 2020) by supplementing 500 or 1,500 FTU/kg in low P diets. However, a positive effect was also observed when using phytase in diets that were formulated to meet the P recommendations (Wu et al., 2004; Amiri et al., 2021) supporting the hypothesis of “extra-phosphoric-effects” by supplementation of high dosages of phytase to phytate-containing diets.

The measurement of IgA showed that increasing levels of phytase led to higher levels of this immunoglobulin. IgA is mostly secreted by mucous membranes, especially in the intestine, and it is the dominant antibody produced in mammals (Hapfelmeier et al., 2010); an analogous Ig is also found in chicken (Davis et al., 1978). Secreted IgA represents one part of the innate immunity of the intestinal mucosa and its presence prevents the adherence of the virus and bacteria to the epithelial cells. The increase in IgA concentration on the mucosal surface and in intestinal digesta due to phytase has been observed previously and may demonstrate an improvement in immune competence (Liu et al., 2008; Islam et al., 2017). Several studies propose, that phytase-mediated metabolites of IP_6_ could be responsible for the regulation of the immune activity (Vucenik and Shamsuddin, 2006; Bozsik et al., 2007).

The possible influence of phytase supplementation on mucus production was also monitored. Goblet cells secrete mucin (MUC)-2, the major component of intestinal mucus (Pelaseyed et al., 2014). MUC-2 is composed of so-called PTS domains, which are rich in the AAs proline, threonine and serine. N-acetyl-galactosamine is the predominant sugar that is conjugated with serine or threonine by the according PTS sequence to form the mucin. Other molecules like galactose, N-acetyl-glucosamine, N-acetyl-neuraminic acid, sulfate groups or more N-acetyl-galactosamine can get linked to the initial sugar for elongating the chain (Paone and Cani, 2020). In the current experiment, no differences in goblet cell counts were observed in the jejunum, and only minimal differences were detected in the cecum due to phytase supplementation. Although similar amount of d-galactosamine was found in ileum content of all treatments, the differences in d-glucosamine may indicate a change in mucus secretion or in the composition of the mucus. However, other factors, like a change in the population of mucolytic bacteria, could also play a pivotal role in that context. A previous study has shown that phytic acid induced increase in mucin production when different sources of pure phytic acid were added to broiler feed (Onyango et al., 2009). Authors did observe differences in mucosal response dependent on the phytic acid formulations used. In the present study no clear effect but indication for increased mucus secretion was observed when phytase was added to a diet containing natural phytate. In contrast, a previous study did not find a difference in cloacal excreted sialic acid and crude mucin between birds fed with non-phytase-supplemented and phytase-supplemented pure phytic acid-supplemented diets (Onyango et al., 2009). The differences might be due to the different sources of phytic acids used in the two studies, or due to the use of different markers (sialic acid and crude mucin vs. d-glucosamine and d-galactosamine) and the different sites sampled (feces vs. ileal digesta). It may also be hypothesized that diet composition in general and other antinutrient factors therein may have a higher impact than phytate and phytase supplementation on mucus-related variables.

To evaluate the absorptive potential of the jejunal mucosa, electrophysiological measurements were conducted ex situ using the Ussing chamber technique. Due to the limited number of chambers only jejunal mucosa of treatments LP1 and LP4 were evaluated. The Ussing chamber´s device sets the electrical potential difference in a tissue to zero by applying a current. The addition of a test substance, e.g. glucose, on one side of the mucosal tissue induces ion transport through the tissue and a change in the potential difference that is compensated by an increase in the Isc for maintaining the potential difference at 0 mV (Boudry, 2005). Thus, the Isc can be used to measure the active transport of substances. The use of phytase induced an increase of the active glucose transport, a result that has also been observed in previous studies (Woyengo et al., 2011; Lu et al., 2020). However, these findings cannot be fully supported by the literature, due to the low number of available studies. Therefore, two possible explanations can be proposed. On one side, the breakdown of the IP_6_ molecule by the phytase releases myo-inositol into the bloodstream. As shown by previous studies, myo-inositol transporters may compete with glucose transporters for sodium, reducing the glucose uptake by the cells (Gonzalez-Uarquin et al., 2020). Based on the results from the current study, we can hypothesize that glucose transporters may have been upregulated in small intestinal cells to compensate for this competition. On the other hand, it can be speculated that phytase supplementation may counteract the negative effect of IP_6_ on glucose absorption as described by Woyengo and Nyachoti (2013).

The Gt represents the measurement of epithelial permeability and finally the integrity of the tissue. Addition of carbachol to the serosal side was used for measurement of the chloride secretion and for monitoring of tissue´s viability during the experiment. The latter was proven in the present study.

Finally, it is worth noting that quadratic effects were observed in most traits, suggesting that responses to phytase supplementation may level off at very high inclusion rates.

In conclusion, the use of increasing doses of exogenous hybrid 6-phytase up to 3,000 FTU/kg of feed in broilers from day 1 to 21 of age improved performance, prececal P and AA digestibility. In addition, supplementation of a phytate-containing broiler diet with increasing phytase dose rates positively impacted some traits related to intestinal physiology, mucosal morphology and immune response, effects that can be called “extra-phosphoric”. However, further studies are needed to confirm these findings and to explore underlying mechanisms in greater detail.

## Disclosures

Peter Ader is employed by BASF SE, 68623 Lampertheim, Germany, which provided funding for this research. The authors declare that, aside from the disclosed affiliation, they have no known competing financial interests or personal relationships that could have influenced the work reported in this article.
